# “Wherever I Go, I Have It Inside of Me”: Indigenous Cultural Dance Narratives as Substance Abuse and HIV Prevention in an Urban Danza Mexica Community

**DOI:** 10.3389/fpubh.2021.789865

**Published:** 2022-01-21

**Authors:** Angela R. Fernandez, Ramona E. Beltrán

**Affiliations:** ^1^School of Nursing, University of Wisconsin-Madison, Madison, WI, United States; ^2^Graduate School of Social Work, University of Denver, Denver, CO, United States

**Keywords:** transnational Indigenous, place, cultural dance, chronic disease prevention, narrative methods, community-based participatory research, substance abuse, HIV

## Abstract

**Introduction:**

“Mexican American Indian” (MAI) is a large and diverse population for which little empirical research on alcohol and other drug (AOD) use and HIV is available, yet for which there is a disproportionate risk. Indigenous health narratives and participation in place- and settings-based cultural practices can be protective in chronic and co-occurring disease prevention and health promotion for Indigenous people. This study explores the role of participation in cultural dance in generating narratives of prevention and health promotion among a sample of MAIs from an Urban Danza Mexica Community (UDMC), framed within a decolonizing narratives of health (DNOH) model.

**Methods:**

This secondary data analysis (*n* = 9) is drawn from a qualitative AOD and HIV health needs assessment of UDMC living in the Pacific Northwest and the Rocky Mountain West (*n* = 21). This study uses a community-based participatory research approach and employs narrative, Indigenized methods to analyze in-depth interviews from adult cisgender females (*n* = 5) and males (*n* = 4). The DNOH model is developed as a relational, analytic framework that contextualizes Indigenous stories in relationship to three distinct yet interconnected levels—the personal, the communal, and Indigeneity in the larger world. These levels of narrative analysis function as culturally grounded, relational pathways through which to articulate health education and promotion approaches.

**Results:**

Narratives delve into the complex and nuanced relationships within participants' internal worlds (personal), between themselves and their Danza community (communal), and between themselves and their complex, intersectional Indigenous identities within society (Indigeneity). Stories of ancestral teachings about health and prevention shared within the Danza circle create spaces wherein participants navigate complex conversations that resist oppressive colonial narratives, reconnect with and strengthen their Indigenous identities, and strive toward ancestral visions of health and well-being.

**Discussion:**

This study contributes to Indigenized theoretical and methodological expansion and the development of place/settings-based, narrative, cultural health interventions aimed at preventing chronic and co-occurring disease and promoting wellness among populations similar to the UDMC. Identifying cultural practices as Native Hubs (relational, socially constructed places) that foster decolonizing narratives helps increase understanding of their role in public health education and promotion through recognition of Indigenous knowledge systems and frameworks.

## Introduction

Indigenous peoples of North America have developed and maintained cultural and community-based health practices grounded in original teachings and knowledge systems that have been instrumental in maintaining health and resilience despite centuries of colonial oppression (1–3). A growing body of health intervention literature documents how Indigenous communities and scholars have successfully collaborated to design and implement chronic disease prevention interventions grounded in such practices (4–9). Most of this literature comes from Indigenous communities whose ancestral origins lie within what is now called the United States and Canada. This body of literature is seminal, with relevant implications to other Indigenous Peoples across North America and beyond. However, there is scarce literature documenting the health status, risk, and protective factors of transnational Indigenous Peoples of North America, who may experience both similar [e.g., racial discrimination, colonialism (10)] and unique [e.g., xenophobia (11)] stressors compared with American Indian/Alaska Natives (AIAN) in the U.S. or Canadian First Nations peoples (12, 13). One such group is Mexican American Indians (MAIs), a new U.S. Census (14) category that includes Indigenous peoples from Mexico living in the U.S. (15). A first-of-its-kind, culturally-anchored, community-based AOD and HIV needs assessment with a sample of MAIs found that AOD abuse and HIV are important health concerns, and that traditional knowledge, cultural practices, and teachings were promising as prevention and intervention strategies. The needs assessment's findings highlighted Danza Mexica (also called Danza Azteca or Aztec dance) as a central cultural practice and prevention and intervention strategy (16). Drawn from the needs assessment data, the present study is an in-depth analysis of the role of participation in Danza Mexica as a potential protective factor and prevention and intervention strategy, framed through development of a culturally-grounded decolonizing narratives of health (DNOH) model derived from participants' stories.

### Mexican American Indians

Mexican American Indians represent the fourth largest Indigenous grouping in the United States, and their population has tripled since 2000 (15). Used as an umbrella term, MAIs include diverse groups of people with complex, intersectional identities who maintain affiliation or attachment with one or more Indigenous Peoples from Mexico. MAIs may self-identify with one or more racial or ethnic identities, may have been born in the United States, Mexico, or elsewhere, and may be U.S. citizens, residents, or undocumented (17–19). MAIs include diverse subgroups constituted around shared cultural experiences and identities (12). These groups may also share a history of Spanish colonization, oppression in Mexican society, and discrimination as immigrants in the United States [specifically non-U.S.-born MAIs (11)]. Spanish colonizers committed multiple acts of genocide and massacre and relentlessly attempted to annihilate their Indigenous ancestors' economic and social systems (20–23) as well as psychological, cultural, and spiritual well-being in order to subjugate the people into indebted servitude and Catholicism (20, 21, 24). Many people were forcefully compelled to adapt, adopt, and exchange their Indigenous traditions for those of the Catholic faith to preserve and protect their traditions from complete decimation. However, many also strategically continued their practices privately within their homes and communities, maintaining their cultural roots while surviving Spanish colonization (25) through the nineteenth-century independence of Mexico from Spain, as well as under continual and increasing Westernization and marginalization of Indigenous People through the twentieth century within the nation state (24).

### AOD, HIV, and Social Determinants of Health

Similar cultural experiences of colonial-based historical and contemporary traumas, such as forced migration and displacement, discrimination, violence, and ongoing structural inequalities (26, 27), are major social determinants of health for MAIs (28). *Historical trauma*, defined as complex, cumulative, and collective traumas resulting from violent colonial acts targeted at specific communities and experienced throughout the life course and across multiple generations (10, 29–31), is a term used to conceptualize these traumas that may act as social determinants of health. Physiologically, historically traumatic events and resilience can become embodied or biologically incorporated, impacting health outcomes and disparities (32) for MAIs (10).

Epidemiological evidence points to AOD and HIV disparities among AIANs and Latinos—two groups in which MAIs may be categorized separately or simultaneously—and the use of alcohol or any other substance is closely associated with increased HIV risk for all populations (33). The AIAN mortality rates due to alcohol-induced causes and drug-induced causes are 6.6 and 1.5 higher than all U.S. races (34), and AIANs have twice the HIV infection and AIDS rate compared with Whites (35). Hispanics who drink are more likely to binge drink compared with non-Hispanic Whites (36), Hispanic males are three times as likely to have HIV or AIDS compared with White males, and Hispanic females are three times as likely as non-Hispanic Whites to die of HIV infection (35). Findings from the first and only needs assessment with a sample of MAIs to date is consistent with these disparities—AOD and HIV were identified as significant community health concerns among 80 and 60% of participants, respectively (12).

This needs assessment not only identified risk factors for AOD and HIV rooted in historical trauma but also found cultural protective factors derived from traditional teachings and practices, one of which was an engagement in Danza Mexica. Danza teachings and practices were described as preventative and promotive of cultural identity, community support, and healing by participants (12). These findings are supported by the literature. Research on dance therapy across non-culturally specific dance forms (e.g., ballroom dancing) and other forms of movement (e.g., tai chi) demonstrate positive impacts on overall health, well-being, and social support (37–39). Cultural dance not only provides mental and physical health benefits for Indigenous and other communities (40–44), but may also increase the likelihood of intervention acceptability, by integrating aspects of mind, body, and spirit, which is linked to motivation and adherence (38).

Furthermore, as an expression of Indigeneity, Danza Mexica may serve as a means to re-create the connection to original tribal homelands and Indigenous identity (45–48). For members of the Urban Danza Mexica Community (UDMC), participation in Danza circles can be a way of cultural place-making to maintain connections to Indigenous identity and facilitate spaces for physical and emotional well-being (12, 24, 48). Native Hubs is a term used to describe relational place-making through Danza participation. Native Hubs can represent fixed or fluid places that may be geographical, socially constructed, or virtual. They can include engagement in a wide range of cultural practices such as pow-wows, storytelling, ceremonies, or other social or political activities that support Indigenous constructions of identity, culture, community and belonging apart from original land bases (16, 48, 49).

### The Danza Mexica Community

The needs assessment sample comprised members of the Danza Mexica community (16). This community is found across North America and the world and is organized around traditional Indigenous dance originating from Mexica (Aztec) peoples, considered the predecessors of Nahuatl speaking peoples of Central Mexico. Danza Mexica communities come from multiple Indigenous Peoples that may or may not include Mexica but who follow Mexica traditions in Danza. In the needs assessment, almost all participants identified unique Indigenous or tribal heritage with only two identifying Mexica as their primary identity. Other tribal groups represented included a combination of Mexica and P'urhépecha, Yaqui, and Aztec. Huichol, Mixteco, Ñuu Savi, Raramuri, Tarahumara, Cora, Otomi, Apache, and Chichimeca were also represented in the sample (16). *Danzantes* (a general term that describes any dancer from the pre-Cuauhtémoc, Conchero, Aztec/Azteca, or Mexica traditions) share a strong history of resilience in the face of traumatic events resulting from Spanish colonization, Christianization, and Mexico's *mestizaje* project (12, 25, 50) as well as on-going discrimination in Mexico and structural inequality and xenophobia in the U.S. (16). These traditions are derived from four evolutionary periods of Danza, starting before (pre-Cuauhtémoc) and after (Conchero, Aztec/Azteca and Mexica, respectively) Spanish invasion through today (24, 25). For pre-Cuauhtémoc danzantes, dance was both a public and private obligation, with its role in prayers and ceremonies related to agricultural events, commemorations and celebrations of human and astronomical events, expression of gratitude, commerce, education, and commitment in social relations (24). Despite Spanish colonizers' attempts to annihilate Indigenous societies (20–22, 24), some danzantes strategically combined their traditions with Catholic rituals, which became the Concheros tradition. Others continued their original practices privately within their homes and communities (24, 25). Into the twentieth century, the Aztec/Azteca and Mexica movements arose and supported more decolonial processes (47). Of the variety of nuanced and dynamic Danza Mexica forms practiced across North America, participants in this sample primarily participate in the Mexicayotl tradition, a more recent tradition centering on decolonizing and Indigenizing practices. While danzantes today practice a variety of health-promoting cultural and ceremonial traditions (e.g., velacion/all-night vigil, temazcal/sweat lodges, and tipi ceremonies) alongside Danza Mexica (12), the present study builds on and deepens the parent study focus on Danza Mexica as a Native Hub (16, 48), in which participation can serve as a protective factor. Using secondary data analysis, it draws from the needs assessment (16) to specifically examine the role of participation in Danza Mexica as potentially protective, through generating narratives of prevention and health promotion, framed by the culturally-grounded DNOH model derived from participants' stories.

## Materials and Methods

### Sample

The first author analyzed nine of the 21 full interviews from the dataset (12). This sample size is appropriate for capturing meaningful data in response to the research question, given the narrative case-based structure for analysis and similarity of the demographic diversity of this sample to the total sample [(51), p. 403]. The first author conducted seven of the interviews alone, and two together with the second author. Six participants were interviewed in Spanish and three in English (one participant occasionally used words in his Indigenous language). Participants were 18 and older [divided into younger (18–39) and older (40 & up) adult cohorts to add an additional layer of de-identification], and education levels ran from zero–eighth grade to graduate and professional degrees. In the study presented here, two participants did not report a particular tribal heritage, and the remaining seven participants identified ancestry from four specific Indigenous Peoples (one of which was Mexica). These Indigenous Peoples have geographic origins across the northern, central, and southern states of Mexico. Five participants self-identified as cisgender female, and four as cisgender male (see Table 1). One participant identified as two-spirit. Two-spirit is an umbrella term that can be used to describe lesbian, gay, bisexual, transgender, and queer (LGBTQ) American Indian/Alaska Native (AIAN) and other Indigenous peoples including participants in this study (52–54).

**Table 1:** Demographics of the participant sample.

**Demographic area**	**Sample characteristics (*n* = 9)**
Age cohorts	Younger adults: 18–39 (*n* = 5) Older adults: 40–60 (*n* = 4)
Genders	Cisgender female (*n* = 5) Cisgender male (*n* = 4)
Interview languages	Spanish (*n* = 6) English (*n* = 3)
Education levels	0-some college <1 year (*n* = 3) Some college >1 year (*n* = 3) Bachelor's-professional degree (*n* = 3)
Indigenous groups	4
Regions of Mexico	3 (northern, central, southern)

### Procedure

This study is a secondary data analysis drawn from an in-depth qualitative community-based participatory research (CBPR) pilot study to gather data to inform an AOD and HIV needs assessment within the urban MAI community of the Pacific Northwest and the Rocky Mountain West (12). The pilot study used a purposive, snowball sampling strategy. Inclusion criteria were adults 18 years of age or older, identifying as men, women, and gender non-conforming (e.g., transgender, intersex, two-spirit, or other tribal-specific gender identity), and those who self-identified as having Indigenous ancestry of Mexico or Central/South America. In-depth interviews and focus groups in English and Spanish were conducted to explore participants' experiences, thoughts, and feelings related to AOD, HIV, and overall health within their communities. For the present study, the first author was invited by the second author (PI) and the project community advisory board to add a place and health theme to assess perceptions of the place relationship in MAI's health. Participants told stories of Danza and place in health throughout the interview question themes, in addition to their responses to the place-and-health set of questions. We took a fluid, narrative-style approach to interview participants that allowed us to follow a non-linear, Indigenized approach to the questionnaire. This enabled the participant's natural storytelling flow to lead the interview. To ensure data collection was responsive to community perceptions/needs, we presented a thematic analysis of the data and the DNOH model to the community for review and approval after data collection was completed (12). While the parent study focused on AOD and HIV risk and resilience perceptions, the analysis presented here focuses on narratives of participant perceptions of health and place relationships.

### Analytic Strategy

Informed by narrative inquiry, the present study uses the DNOH model as an analytic framework and narrative thematic analysis (NTA) (55) as the specific analytic strategy used to identify story segments for analysis and interpretation of the data within the DNOH model. Aligned with Indigenous, relational worldviews, narrative inquiry posits that understanding of the whole sheds light on understanding the parts and uses the analysis of stories to explore and create meaning (56). This study conceptualizes participation in Danza as a Native Hub (48, 57), to explore the potential impacts of Danza participation on AOD, HIV, and other overall health-related prevention goals, which in turn expands the reader's knowledge of how such practices may be transferrable to similar situations (58). It also functions as a tool to move beyond theme identification into an analysis of the themes' interrelationships to uncover more nuanced processes within the context of conceptual ideas (56). Narrative, like storytelling, is also a primary medium of communication among many Indigenous nations and is an effective method to assess determinants of health (59).

This study uses the DNOH analytic framework to help guide the analysis and interpretation of the data. The DNOH model is inspired by the Kaupapa (Māori language: principles) Korero (Māori language: to talk, narrative) narrative analysis method (60, 61) as well as Danza Mexica epistemology (24). It delves into the complex and nuanced relationships within participants' internal worlds (personal), between themselves and their Danza community (communal), and between themselves and their overall Indigenous identity within society (Indigeneity)—contextualized within Danza as a Native Hub. Using the DNOH framework aligns with the circular formation of Danza, like a solar system within a universe, both a reflection of life and connection with Mother Earth (24).

The transformative role of Danza as a vehicle for decolonization can also be depicted by likening the DNOH to the structure of the Danza circle. Each danzante is like a planet that strives to move in balance and duality with other danzantes within and across the circles, honoring *Ometeotl* (the Nahuatl word often translated to mean “dual energy” also referred to as Higher Power, Great Spirit, or Creator/God, as dual feminine and masculine energies in one force to create life; sacred or divine duality) in word and action (47). Most novice dancers start on the outer rings of the circle and move closer to the center as they increase in knowledge, skills, and responsibilities within the Danza community—similar to the process of UDMC strengthening or returning to their identities as Indigenous people through decolonizing narratives while simultaneously remaining grounded in their Indigenous identity within the larger society.

Furthermore, the DNOH model demonstrates how knowledge, health, and decolonization is transmitted intergenerationally, through the Danza circle structure, in that those danzantes closer to the center function like elders, or more experienced persons who help the novice danzantes grow in knowledge and responsibilities of their own Indigenous identity (24) and within the group. No matter where danzantes find themselves within the actual Danza Mexica circle or where individual narratives within this study are located within the DNOH analytic framework, each person plays a unique and equally valuable role that helps the entire group function as a *calpulli*—like a family [Nahuatl notion of a group; (46) p. 119].

**Figure 1:**
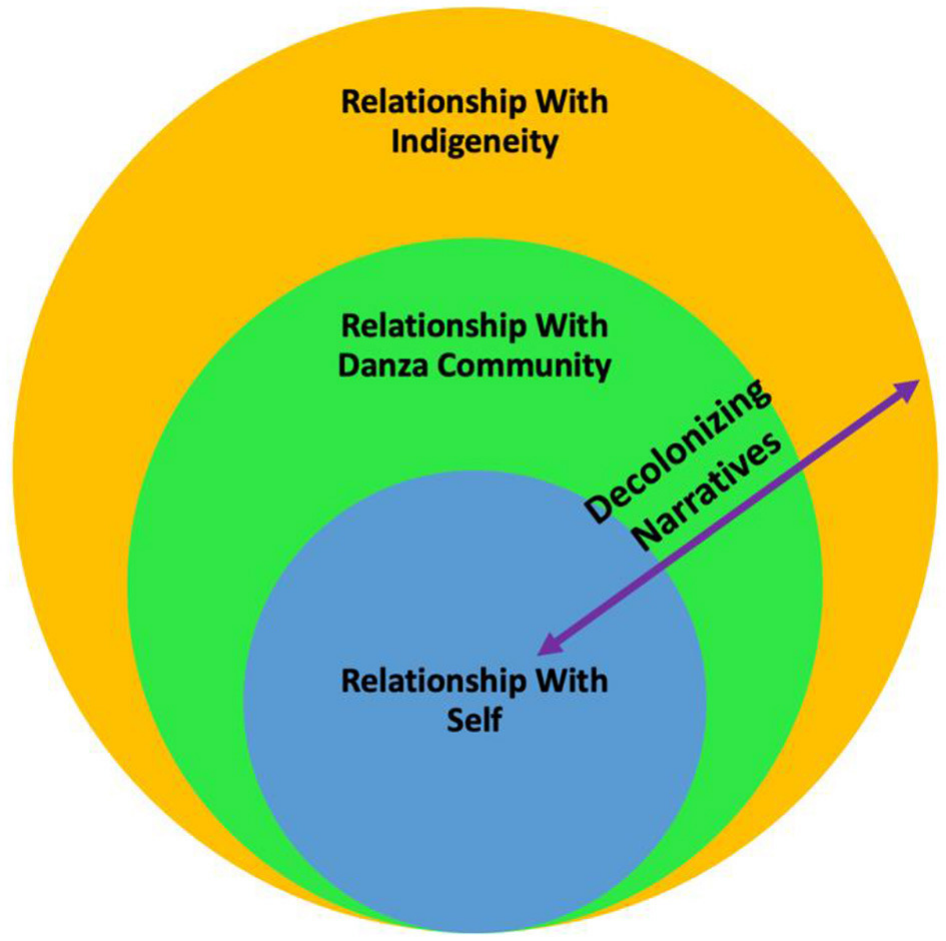
Decolonizing narratives of the health analytic framework.

The DNOH model (see Figure 1) in its entirety represents the Danza circle as a Native Hub (48) in which participants' stories function as decolonizing narratives (represented by the left-right double arrow) of connectedness to self, others, and Indigeneity within the larger society—narratives that may transmit Indigenous teachings of health and well-being. The three layers of relationships within the DNOH model—self, Danza community, and Indigeneity— are inextricably linked through the concept of connectedness. *Connectedness* refers to the interdependent well-being of self, family, community, and the natural environment (62), as well as the role the concept plays among Indigenous people in creating cultural places that are optimal for prevention and health promotion (63).

This study highlights this role for the UDMC as an urban, transnational diasporic community that sustains their overall connectedness to place—representing their ancestral origins and attachment to homelands—through participating in Danza as a Native Hub. The first layer, relationship with self, narrates Danza's role in place-making for participants' connectedness with their own emotional, mental, spiritual, and physical health. The second layer, relationship with the Danza community, narrates Danza's role in place-making through fostering health within the context of their connectedness to Danza as a family or community structure. The third layer, relationship with Indigeneity, narrates Danza's place-making role for cultivating health within the context of connectedness to their identities as Indigenous people in relation to one another and other groups in the larger society. The left-right double arrow that crosses all three layers represents how participants use narrative as a tool with which to decolonize beliefs and practices surrounding prevention and health promotion, engaging in Danza as a Native Hub that facilitates traditional health beliefs and practices, thereby “weakening the effects of colonialism” [(64), p. 65]. Such traditional health beliefs and practices may include (but are not mutually exclusive) community health-promoting traditions such as velacion/all-night vigil, temazcal/sweat lodges, or tipi ceremonies in addition to Danza Mexica. These practices are also integral to connectedness to Indigenous identity, and may additionally be practiced by danzantes across multiple levels of the DNOH. However, the present study is centered on Danza Mexica as a Native Hub within which traditional health beliefs and practices are engaged. The layers of the DNOH are not mutually exclusive in that participants often traverse within and across practices and layers several times within a single story.

Finally, we used NTA as a way of organizing and making meaning from the data about the role of Danza in participants' relationships to place and health. The primary purpose of the NTA in this article is to identify key participant stories relevant to the research question to highlight and magnify each participant's voice. The NTA emphasizes data content—what participants say—with the intact story as the unit of analysis from which to theorize, rather than smaller clusters of words or phrases (55, 65) through exploring the meanings within the stories as individual cases. The NTA can accommodate the large amount of data generated through qualitative data collection, making it a strategic foundational step that provides narrative material from which the remaining analytic strategies can be drawn.

This NTA generates themed, categorized stories from participants' narratives that draw on central concepts of the ISCM and highlight individual and collective meanings of UDMC experiences with health, place, and dance. These stories are the foundation for the DNOH analysis. The NTA was a preparatory mechanism used to identify and organize stories to conduct a more nuanced analysis. The first author developed the DNOH model to collate the stories and integrate them into an analytical framework of layers of contextual relationships to examine these connections across levels for each participant. The first author followed five steps: (1) summarization of participant background; (2) guided selection of story segments by the research question and DNOH layers; (3) summarizing interpretation of main story points; (4) using the DNOH model to guide placement of stories in layers; and (5) examining stories within and across themes and patterns (61, 66, 67).

**Figure 2:**
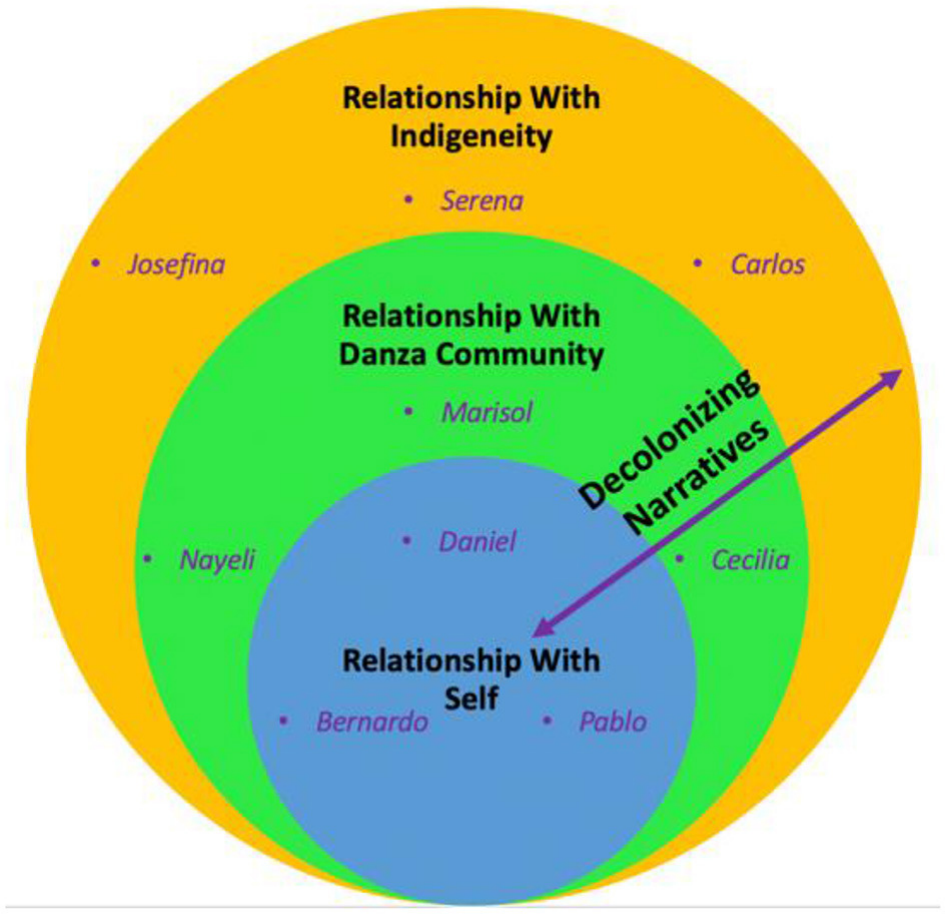
Decolonizing narratives of the health analytic framework: placement of participant narratives.

Overall, our data analytic approach guided the selection, placement, and interpretation of these particular, salient stories within each participant's narrative. Each participant's story was treated as a case within a particular layer, in alignment with the methodological design, which favors lengthier excerpts over more brief, fragmented excerpts, to preserve interpretive essence better and serve as appropriate units of analysis [e.g., (55, 61, 65, 68)]. The section Results is organized according to the three DNOH levels, each containing the participant name (pseudonym) whose narrative is highlighted (Figure 2).

## Results

Throughout these stories, participants engage both individually and collectively in the process of decolonization. The first author found an overarching theme of Danza as a Native Hub—a place wherein narrative is used to decolonize conceptions of identity, health, and well-being through the transmission of traditional health knowledge within the context of individual and collective Indigenous identities. Conceptualizing Danza as a Native Hub, specific subthemes embedded within the three layers of the DNOH model illuminate the pathways through which participants achieve the overarching theme. These include spiritual, mental, physical, and behavioral health and well-being, sense of family and belonging, community education and advocacy, and Indigenous identity and solidarity.

### Layer 1: Relationship With Self

#### It Sets a Blueprint for You

Daniel, Bernardo, and Pablo tell the story of their personal journeys in confronting depression and anxiety, physical pain, addiction, and both internalized oppression and discrimination based on race. Each of their narratives portrays Danza as a space of healing whose teachings help them navigate their specific life challenges. They speak of embracing their Indigenous identities and unique roles within the *calpulli*, embodying the teachings of their own stories to honor their ancestors through self-love as well as through dedication to preventing illness and promoting health among future generations.

##### Daniel (Younger Adult Male)

Daniel's story frames Danza as a spiritual journey that provides a pathway through which members of the UDMC can find healing from mental stress through spirituality and Indigenous identity. Daniel describes Danza as a personal “outlet” to find his “inner peace,” rooted in ancestral origins.

You could go to Danza, and you can dance, and during that time... you can do some self-reflection... exclude yourself from the outside world and just find a connection with yourself... It's just another outlet for people to feel at peace with themselves.... You could do that with going to church, or... a temple. The differences is our ancestors.

According to Daniel, Danza “keeps a lot of people healthy,” not just physically but also spiritually. For danzantes, he claims, Danza's teachings “set a blueprint for you to carry on those things through your everyday life.” His story exemplifies that narrative helps danzantes find a sense of balance. The ceremonial practice of using *palabra* (“the word” in Spanish), a ritual process within which participants are given the opportunity to express themselves from their “mind/heart” [(24), p. 103]. This sacred process within Danza, usually enacted at the end of ensayos (practices) or formal ceremonies, serves as an expressive outlet through which one may find support for mental health:

When we finish... we give everybody the opportunity to speak…one of the things that we focus on the most, is people being mentally healthy because there will be times where someone will feel like they want to give up; and so our group really focuses on trying to help them out... whether it be an elder or someone who has a high position that can help them out and talk to them... when it comes to mental health, I think that Danza is a very big pillar to my community.

Daniel's narrative speaks to the role of spirituality in Danza as a Native Hub to heal, transform, and empower oneself. Such spiritual teachings provide a guide for living a healthy life. According to Daniel, the spiritual consciousness Danza brings provides a sense of purpose and identity for both him and his community.

##### Bernardo (Older Adult Male)

Bernardo's story exemplifies the power of Danza as a decolonizing Native Hub wherein he learns Indigenous Danza teachings that his mother forbid as “despicable” during his childhood, giving him the spiritual strength to overcome addiction, regain a sense of self-worth and trust in others, and a sense of purpose. Now a dedicated member of a Danza circle himself, he shares that engaging in Danza weekly strengthens his spirit to handle the stressors of daily life. He reflects on the importance of his wellness in being able to support loved ones both in the United States and Mexico:

Danza has also helped me quite a bit; it gives me spiritual strength. Asking for blessings to begin the week right, for our people that are over there and those that are here. When people depend on you, you have to be strong and take care of yourself.

Danza is a holistic, healthy space, according to Bernardo. It has “helped me to give up alcohol, drugs, to put into practice the knowledge of what to do with our lives,” and helped him learn “how to live”:

…the emotional problems that are most difficult. I was tired…but when I heard the drums…I arrived at my safe door…I can feel things differently…when you use drugs, you feel like you are worthless…when you begin to detoxify your body…you recuperate… to revive your trust in people…to believe in something…this type of health, this type of group…is what many people want…to return to feeling that you are alive, that you begin to act like a human being again.

Bernardo's story serves as a testament to resilience and strength in healing oneself to be a healing force for others. He credits Danza for playing a crucial role as a Native Hub wherein he has overcome addiction and wherein he came to embrace his Indigeneity. The passion he expresses for Danza's role as a place of healing in his own life translates into a dedication to promoting health and pride in Indigenous identity among youth and others within his community. Bernardo's words embody this mission for his people: “I keep identifying myself as a Mexican and as an Indigenous…wherever I go…I have it inside of me.” Danza as a Native Hub is the place wherein his reconnection with ancestral origins is cultivated.

##### Pablo (Older Adult Male)

Like Bernardo, Pablo's story exemplifies the power of Danza as a Native Hub wherein he also has learned to cope with mental and physical health challenges. He has overcome addiction through learning traditional teachings about staying healthy, which give him hope for the future. He reflects on the initiation of his healing journey with family in Mexico and his Danza family after he arrived in the United States:

I had a threat to my health, emotionally…I began to suffer from anxiety attacks. It was when I told the doctor that I…I began to live with these anxieties. I sought out someone who could listen to me because I had a lot of things going on…and I entered into a depression. And my [specific family members] helped me so much…speaking with me. They took care of everything. I started going to see psychologists, and I learned a lot from them…. I was going to therapy daily…and this was when they told me about [support group for addictions]…and they helped me a lot…. I began to take out all of my frustrations…and my anxieties began to lessen, and so did my blood pressure. I came here, and I had this depression, and through Danza…I have learned how to endure it.

Grounded in his culture, Pablo talks about the traditional teachings and holistic health benefits of Danza participation that make his pain “vanish.”

[F]or us, it is medicine. We should be eating well first because we have to be healthy, this is the base of everything…. And after this, we utilize Danza…when we are physically unwell, we use traditional medicine…but we also utilize the spirit of the Creator…. We ask that he help us with Danza, if we are physically unwell…the medicine enters through the spiritual, and it enters through the physical. But before all of this, we ask the spirit…we arrive with pain, and when we are dancing, it all vanishes.

Since his arrival to the United States, he explains that Danza has provided a healing and empowering Native Hub. Therein, he finds holistic “medicine” to support his recovery from addiction and physical and mental health conditions. Pablo's narrative tells how he has learned to become a healthier person through traditional teachings and Danza's practice.

### Layer 2: Relationships With the Danza Community

#### We Take Care of Each Other

Marisol, Nayeli, and Cecilia tell the story of Danza as a Native Hub that fosters a sense of family and community, a *calpulli* through which they give and receive social support to face health challenges, addiction, and emotional stress. Each of these participants traverses unique and challenging territory as they seek and create a sense of belonging within (and at times beyond) the Danza community. Within this support system, participants share their knowledge, skills, and advocacy in risk prevention and health promotion, making the Danza *calpulli* into a place where “warriors”—young and old—can emerge.

##### Marisol (Older Adult Female)

Marisol's story reflects the central role of family that Danza as a Native Hub creates and the role of advocacy and outreach in creating community. She explains how the sense of family connectedness cultivated within Danza can create a sense of belonging, purpose, and identity that can help danzantes overcome addiction and mental illness and promote physical and mental wellness:

…those that have gotten involved in the group…the benefits of belonging to a group, a *calpulli*, a family, to transform their lives and leave behind alcohol and drugs, to have a purpose in life and identify with knowing where you come from, knowing that you have a past and that you have a group that has your back and makes you feel that you have a future.

Marisol emphasizes the important role of advocacy for the prevention of health risks through passing on traditional knowledge and health practices to young people as the “seeds” of the future. She and her fellow danzantes provide outreach to the Mexican-origin community, expanding Danza as a Native Hub to welcome others beyond their circle. At school and community events, they present as a family, sharing messages of prevention and health promotion, awakening consciousness and pride in Indigenous identity through connectedness to one another in their common ancestral roots. Marisol's description of the group's message emphasizes the key role that Danza plays as a protective factor from stressors—a Native Hub through which connections to community, homelands, and identity are maintained wherever they go.

[W]hen we give presentations, as a group, as a community, as a *calpulli*, what we bring isn't just Danza, to the schools, the community events, what we bring…is…the image of Danza and what the culture is. And it makes them feel…that you are not alone. That they have this identity that even though they are living here in this country, they see themselves reflected in the Danza group, their past, the family that they left in their countries, or in Mexico…. That even though they are far from their country, they can as a community keep recovering their values and traditions…medicine, the food, the traditions as well as the culture. That they can keep maintaining the unity of the community…to the children in the schools that suffer from bullying, that suffer from discrimination, that they see that the culture of their family, of their parents and grandparents, with this traditional garb, this coordination and these dances, it gives them, it awakens their pride. Which is to say they are my culture or the culture of my father or mother or my grandparents. It is this connection that awakens them…. I belong to this community, I belong to this town, to this culture, to this tradition.

Belonging extends beyond collective gatherings. For those who cannot attend a Danza circle, Marisol describes Danza as a transportable Native Hub: “And if you are not able to go to Danza, you have your altar in your home…all of this heals you.” Marisol's story portrays that connection to Danza as a Native Hub can be expansive, fluid, and flexible beyond the Danza circle itself, to the home or community, facilitating a commitment to caring for self and community no matter where one goes: “is healing, it is medicine.” Ultimately, Marisol's story is an example of how Native Hubs can help participants stay connected to spiritual well-being no matter where they are, through sharing its healing message of belonging with all MAIs and their descendants—the “seeds” and “future” of her community.

##### Nayeli (Younger Adult Woman)

Nayeli's story exemplifies how Danza is an ideal Native Hub for fostering a sense of *calpulli* (family), especially for youth. Within Danza, she shares her journey of reconnecting with and embracing her Indigenous heritage, giving her a sense of belonging that serves as a platform for developing coping strategies for stress management and sharing ideas about prevention and health promotion among youth. Her story begins with her search for a positive community with which to connect, as part of her motivation to join Danza, whose teachings have taught her “how to cope with myself.”

Nayeli shares creative ideas for how her *calpulli* can facilitate dialogue on many health-related issues through intergenerational communication. She suggests one-on-one mentorship and advocacy, from older to younger danzantes, regarding such “sensitive subjects” related to health. For example, Nayeli shares that if she is “having this issue and I needed someone to take me to a clinic, they could take me. They might want to sit me down in front of my mom and talk about it, but they'll always take me.”

For AOD and HIV education, Nayeli gives specific recommendations for organizing small groups or one-on-one meetings, which has been helpful for her in facilitating more effective communication and support:

[M]aybe they have questions that they don't really have answered. Say, we're, like, after practice. We can sit down and, like, whatever's on their mind, we can then talk about; and, say they're having an issue drinking, we could tell them, like, what it is going to lead to, what could potentially happen…what the alcohol is actually meant for…like the spiritual meanings behind it.

Nayeli also expresses her belief that leaders should be educated about other health-related topics like mental and health and interpersonal violence:

It could just be any topic…spiritual needs, substance abuse maybe, they're in a[n] abusive relationship, they're…maybe it's like me that I need emotional support, they can then tell me why I'm feeling a certain way. They can find out the reasons for what triggered it and help me come out of it.

Nayeli's story demonstrates how Danza as a Native Hub on the community level has provided her with not only a sense of belonging within her individual *calpulli* but also within the larger Indigenous community. Within the positive, familial space of the Danza circle, she learns about her history and culture, develops coping skills that help her achieve her goals, and shares her ideas about prevention for youth. With the support of her *calpulli*, Nayeli's story exemplifies how Danza is a Native Hub for fostering leadership.

##### Cecilia (Older Adult Woman)

Cecilia's narrative speaks of the familial space that Danza provides as a Native Hub wherein she plays a role as a protector, nurturer, and healer in educating young “warriors” to avoid substance abuse and gang involvement and mentors them toward health leadership. To facilitate this positive space for healing and growth, Cecilia explains her sense of responsibility as a role model, “pure of my mind and of my heart,” through choosing not to use alcohol and pursuing spiritual growth—in alignment with her Danza circle's values as “a spiritual group.”

Cecilia provides an example in which she chose permanent abstinence from alcohol to make a statement as a role model for her community—her decision based on Indigenous traditions. Her story demonstrates her dedication to taking care of herself, her family, loved ones, and fellow danzantes by assuming the role of protector and healer for youth, families, and other women. She believes that this is part of her role as a woman—to support the emergence of healthy, sober young “warriors” from the group.

I want that my heart and my prayer, because my heart and my prayer are what I give in the Danza, for our people, for our children, for my [partner], for my family in Mexico, for our women, our children, all of the little ones that don't know the situation we are in and how it is changing the world, no? And so for this reason, I am on this path…if in some way I can save a child from getting into drugs or help a mother to not fall into depression because her husband abandoned her or because she doesn't have anyone to watch her kids, for these reasons, we are in the Danza group. To help our women. I feel very proud because we are protecting our community. It is a big responsibility to be taking care of this whole family. But we take care of each other. I believe that good warriors are going to come out of this Danza group, and more little warriors are going to come out of it, from these men and women that we are educating well, to not use drugs, no gangs, no alcohol.

Cecilia's narrative depicts Danza as a Native Hub for community-building—a large intergenerational family where danzantes can feel a sense of belonging and protection—and a place for prevention, health promotion, and education.

We are supporting not only our community but our children. …I feel that we put a lot of energy into it, and we do the best that we can to bring our people in. For many of our youth, it is very important to keep them in Danza, to give them this guidance, education, that having Danza in your life…you're not going to get in trouble because all of us elders, since I am the [order in age group] in the Danza group, we all protect them, we are all their mothers, their fathers, for these young people to the youngest ones, it is a blessing to have the children there, even more so when we have the women with their babies in their womb still, and they hear the rhythm of the Danza, and for this reason, we dance in the schools, in the community spaces.

The overarching theme in Cecilia's narrative is her role in cultivating a sense of family within Danza as a Native Hub. Her story reflects her pride in being a role model of traditional health teachings to play her role as a woman to love, heal, protect, and support her Danza family. She particularly emphasizes her efforts to make Danza a positive space for youth—both in preventing substance abuse and health promotion through pride in identity, belonging, and connection.

### Layer 3: Relationship With Indigeneity

#### We're Always Connected to the Universe and the Galaxy and Our Ancestor[s]

Josefina, Serena, and Carlos tell how, within the Native Hub of Danza, they reconnect with their identities as Indigenous people in the world, finding pride and a sense of solidarity with other Indigenous communities. Through decolonizing narratives, they not only learned coping strategies but also ancestral teachings that enable them to confront the structural oppression that drives many of the health challenges among their people. Within the Native Hub of Danza, they are positioned as leaders—from emerging to seasoned—to resist oppression, build community among other UDMC and Indigenous peoples, reconnect with Mother Earth, and thrive.

##### Josefina (Younger Adult Woman)

Josefina's story documents the beginning of her journey as she finds healing and connection to her Indigenous identity through her engagement in Danza as a Native Hub. She reflects on her new consciousness of and pride in her Indigenous identity here in the United States as well as her initial dissonance and disconnection with Indigeneity while living in Mexico: “At first, it caused me conflict or harm…I am still learning…about defending my roots…. I didn't know my culture…and now that I am beginning to see it, it really interests me….”

Josefina recalls witnessing discrimination toward Indigenous people in Mexico for speaking their languages, and she reflects on her disconnection from Indigeneity at that time. Danza as a Native Hub has provided a space wherein she has begun to explore her identity and express her desire for a greater understanding of her Indigenous roots:

In the people with whom we can share what we really are, what it feels like to be proud of what we are. And so that they can learn to value the things that one can make possible. And that we shouldn't devalue people because they don't speak a dialect…. I think that we are like this when we do not know; sometimes, we judge people who speak a different dialect or because they are different.

Josefina also shares observations about how ceremonial practices tied to Danza support recovery from addiction—something she has witnessed personally within her family. She speaks of how balance is learned through the ceremonial use of Peyote as part of recovery.

… they come to the group because they want to avoid this, and for example, using Peyote…they see it as a medicine. And if really they don't need it, they don't consume it…a ceremony is a question of if one earns it…this requires time, and I think…it helps them a lot. And the discipline that one is learning is, they start distancing themselves from all of this. Peyote, when they use it like this, they try to make sure that people don't confuse it with a drug. And that they don't make it into a necessity.

Within Danza as a Native Hub, Josefina has learned about the spiritual use of Peyote used in specific ceremonies as a respected plant relative. Based on what she has learned, she suggests those struggling with addictions be invited to participate in Danza to experience the healing that comes from dancing and the teachings on connectedness with Mother Earth: “I would invite them to come because the simple act of dancing helps a lot. To feel oneself connected with the earth, with the environment, this has helped me a lot personally.”

Josefina's story is a testament to the power of Danza as a Native Hub to foster decolonization of colonial beliefs about Indigenous inferiority, to question colonial narratives, and to foster healing and connection with ancestral roots. Not only does she share how Danza participation gave her a sense of pride in her identity, but also how it has helped educate her about healthy relationships with substances, their connection with Mother Earth as plant relatives, all within an Indigenous worldview.

##### Serena (Younger Adult Woman)

Through Serena's participation in Danza as a Native Hub, starting in Mexico and now in the United States, she shares how her pride in ancestral roots and connection to Mother Earth continues to grow and strengthens her resistance to both internalized oppression and structural oppression toward of Indigenous people of Mexico. Her story exemplifies that whereas Danza and other Indigenous ceremonies often originate in specific geographical locations, over time, they may evolve and migrate, as do many Indigenous peoples. They may be transported to urban spaces and shared across national and transnational Indigenous Peoples, yet always maintain songs, practices, and teachings derived from their connectedness to Mother Earth and all creation.

[I]n Mexico, many tourists try to encounter…cultural aspects of the traditions, while the…Mexican people do not value it, and we try to avoid it. Or we do not recognize that we are natives, that we are Indigenous…even in Mexico not only here, people…aren't proud to be Indigenous. They prefer to hide it, but there are things one cannot hide …. So, instead of speaking to the government and the institutions telling them to support them and telling them that you are Indigenous and this is important, that you have knowledge, you have tradition, they try to make them less valuable…as if it had less value, that I am Indigenous…and my way of being is entirely the opposite. I am super proud of being Indigenous, and I show it…speaking about the cultural Danza group that we are in, we are all proud, and the people see that we are proud.

Although the UDMC as a Native Hub is far from participants' diverse, geographical homelands from which Danza originated, each Danza remains connected to these lands. Danza practices and ceremonies are packed with multiple teachings on how traditional health knowledge promotes healthy nutrition among danzantes, as part of a relationship of reverence to Mother Earth for all she provides. Serena explains:

All of the dances have something to do with the elements. *Tonantz*í*n* is the name that we give to our Mother Earth…that provides our nourishment, that enabled us to walk. The earth is where we come from and where we will return…when you are in the circle of Danza, you learn to recognize the importance of all living things…you don't take more than you need…they have arrived to cure themselves there, and they give up drugs or alcohol or other things that they use because they are not necessary…there are foods that are not good for you and your body knows it, and somehow it communicates to your mind, and you just don't want it anymore…. And you want to just do all-natural things, and you want to grow your own garden …. You feel that connection with the earth.

Serena's narrative demonstrates how Danza as a Native Hub brings her “home” to her Indigenous identity, no matter how far she now lives from her homelands. According to Serena, Danza is a place of health that grounds her in her Indigenous identity. It enables her to be part of a larger group of Indigenous peoples within and beyond the Danza community. Within Danza as a Native Hub, Serena speaks of learning traditional health knowledge that guides her choices about substances and foods in reverence of her relationship to Mother Earth.

##### Carlos (Younger Adult Man)

Carlos's story exemplifies how Danza as a Native Hub can be used as a decolonizing tool to reconnect to Indigenous identity and strengthen solidarity among people with Indigenous ancestry of Mexico. Born in his Indigenous community's homelands in Mexico, his connection to his Indigenous identity was disrupted: “…because of NAFTA [North American Free Trade Agreement]…we were forced to move out of our village,” making it “hard for us to practice our language.” Like many other Indigenous and mestizo Mexican families, Carlos's family could no longer afford to produce their own corn, driving them to northern Mexico to work in agribusiness, where “it's about exploitation,” and later to the United States where they continued to work in agriculture. Carlos shares how joining Danza in college helped him peel back layers of colonization to return to his Indigenous identity in college:

You got to understand where you're going, where your people stand in order to keep going and fight for what is right for you and your people. And so [danza] philosophy is all about reconnecting to Indigenous roots. That's when I started identifying myself more as [Indigenous]. I was doing more research, talking, introducing myself now as an Indigenous person. Before then, in high school, it was just…a Mexicano…

Carlos's story demonstrates how joining Danza was the catalyst for helping him re-embrace his Indigenous identity. As a transitional Indigenous migrant youth, he experienced racial discrimination first in Mexico and then in the United States, facing pressures to conform with nationalist identities in both countries, yet considered “not Mexican enough” or “not American enough.” He was “changing my identity now…from [name of participant's Indigenous group] kid to Mexican American to…Chicano.” He speaks of his commitment to passing on his Indigenous identity to his future children: “I have to pass on to…my next generation, and make sure… we go as deep as…we can in the culture.”

Carlos's narrative relates a cultural dance specific to his Indigenous group to Danza. He highlights the connectedness that cultural dance, in general, brings to intergenerational, ancestral, and intergalactic relationships, facilitating a sense of balance that ushers in physical and emotional health and well-being.

Like the spirit or yourself fills with proudness and happiness…in [name of Mexican province] we danced…[name of local cultural dance]…. Every time we…dance [name of dance]…we shout, we scream…happiness…whenever you dance, you're doing, like, exercise…We have to have like music…we hold proudness. We love ourself…. It's like a healing of us…we just feel the moment…and feel our bodies…. And relating that to health, I feel like that's how our people [have] always done it…. We dance to fulfill ourself spiritually and emotionally, and also to learn…about balance…with Danza, we always start on the left—because that's our heart. We always close the circle. We open the circle too. And we're always connected to the universe and the galaxy and our ancestor[s], and so that's a very deep-rooted thing.

Carlos's narrative speaks to Danza participation as the catalyst that initiated his journey toward peeling back the layers of nationalist, colonially imposed identities. Through sharing songs, dance, stories, and teachings with other people in the UDMC and even within the larger Mexican community, Carlos speaks of his connectedness to his original tribal heritage as a gift he will pass on to his future children.

## Discussion

Across all nine participants' narratives, each layer of the DNOH model contained important themes that speak to the multilevel role Danza can play as a Native Hub in risk prevention and health promotion. Within Layer 1 (relationship with self) of the DNOH model were narratives of how participants found strategies to manage individual spiritual, mental, behavioral, and physical health challenges through their participation in Danza as a Native Hub. Narratives in Layer 2 (relationship with the Danza community) spoke to collective issues of belonging, nurturance, and protection, both within and beyond the Danza circle, as well as community-level health education and outreach—demonstrating the role that the UDMC plays in the health of the larger Latinx, Mexican, and Indigenous communities. Finally, Layer 3 (relationship with Indigeneity) centered on reconnecting with and embracing Indigenous identity as resistance to Indigenous erasure in solidarity with other Indigenous Peoples and within the context of the larger society, through narratives that remind us of the origins of Native Hubs—as connections to ancestral lands, synonymous with connections to Indigenous identities.

Native Hubs are created and transported wherever Danza is practiced. Narratives are used as specific decolonial tools with which participants reconnect, remember, and recreate traditional health practices and beliefs at the individual, communal, and societal levels. For members of the UDMC, Danza participation is making place—a Native Hub away from ancestral homelands. Living in urban spaces as largely diasporic, transnational populations enables them to maintain their land-based ancestral ties through decolonizing oppressive, colonial narratives within Danza as a transportable Native Hub that they can take with them wherever they go. Within this Native Hub, the UDMCs honor their ancestral origins and the lands from which their identities are borne, and they are charged with setting the stage for future generations to thrive.

Beyond demographic differences, each of these nine participants represents a diversity of lived experiences that shape their perceptions within and beyond the Danza circle and enrich the findings of this study. Across unique differences, narrative served overall as a decolonizing mechanism. Participants shared how Danza has created a Native Hub wherein healing and returning to individual and collective Indigenous identity is nurtured. This Native Hub provides a place of healing for body, mind, and spirit, as well as for finding a sense of belonging and community for members of the UDMC, whether U.S.- or Mexican-born, living in the United States far from their ancestral homelands. Furthermore, it is a place where consciousness is raised, stereotypes and racism are challenged, resisted, and debunked, and intergenerational wisdom, healing, and liberation are transmitted (69).

This analysis represents the highly complex, intersectional, and nuanced experiences of nine diverse individuals with a common confluence of lineage and culture, whose resilience speaks volumes to the power of Indigenous people to survive and thrive despite structural oppression created by settler colonialism. The challenges that Danza groups face now are not only the health disparities and inequities that are common to many other marginalized groups in the larger society. Countering the racist stereotypes imposed on their ancestors, as well as contemporary Indigenous communities in Mexico, participants share stories of traditions that their elders had to hide to keep alive. Identifying Native Hubs as protective places that foster distinct pathways for decolonization helps increase understanding of how they can serve as optimal, culturally grounded place/settings-based interventions for preventing health risk behaviors and promoting overall health and well-being. Similar to the findings from the MAI needs assessment parent study that 80% of participants identified AOD as a significant risk factor and 60% perceived HIV to be a heath concern, the sample in the present study spoke more frequently of concerns about AOD than HIV risk. Furthermore, the present study builds upon and deepens the original MAI needs assessment through its development of the DNOH as a general health model which not only encompasses potential for prevention of AOD and HIV risk, but also includes additional protective benefits such as improved mental health, physical activity and nutrition.

### Practice, Research, and Policy Implications

An AOD and HIV community needs assessment with a sample of Mexican American Indians found that AOD and HIV are important health concerns and that traditional knowledge, cultural practices, and teachings were instrumental to prevention and intervention (12). Using secondary data analysis, the present study draws from this needs assessment to investigate the role of participation in Danza Mexica as a potential protective factor in generating narratives of prevention and health promotion, framed by a culturally-grounded DNOH model derived from participants' stories.

The findings suggest several implications for practice, research, and policy. First, the results of the analysis point to the need for practitioners working with UDMC (or similar populations) to include questions about client participation in cultural practices in their assessments. Should clients express interest or participate in such practices, practitioners should support client engagement and include such culturally grounded interventions in developing treatment plans. It is crucial that practitioners recognize that “many of the culturally grounded approaches proposed are actually a return to traditional worldviews and practices that were part of everyday life for Indigenous communities and a source of their well-being prior to Western intrusion” [(70), p. 250]. Native Hubs like the UDMC in this sample may also serve as places for community organizing and social movements—which may be included as part of a client's treatment plan, based on that person's priorities and interest.

It is also crucial that health care practitioners strive to remain conscious of the great diversity among Indigenous Peoples, their distinct affiliations, definitions of culture, and proximity to particular identities and practices—important factors unique to each patient that will help shape assessments, treatments, and intervention. The results of this study's analysis are particularly crucial to populations similar to the UDMC. The UDMC is a highly diverse population in terms of language, Indigenous grouping, national origin, immigration status, gender, sexual identities, and lived experience.

Researchers working with populations such as the UDMC should take a similarly supportive, engaged, and collaborative Indigenist approach to their work. This approach is found within the body of critical theories of race, intentionally de-legitimizing racism in research and promoting liberation through self-determination and empowerment. It is based on three related, foundational principles: resistance as a tool for liberation (i.e., Indigenous resilience and empowerment despite colonization); political integrity (i.e., research by Indigenous scholars); and privileging Indigenous voices (i.e., research with Indigenous participants); (71). Researchers should support Indigenized methods to privilege Indigenous voices that tell stories of resilience despite the multiple, multi-generational forms of structural oppression within which they live.

This study exemplifies the use of such Indigenized methods to uncover opportunities to expand the current literature on culture- and place/settings-based health interventions. Within Danza as a Native Hub, we learn that decolonizing oppressive colonial narratives through reconnecting with ancestral teachings and practices help danzantes learn, internalize, practice, and pass on knowledge and strategies for risk prevention and health promotion at individual, communal, and societal levels. In a sense, by sharing and maintaining these threads of attachment to homeland/Indigenous identity in narratives, identity is kept alive. It can thrive no matter where Indigenous people go. Taking the lead from the participants' use of narratives as a decolonizing tool, this study enhances mainstream understandings of place/settings- and culture-based interventions within public health and other related fields by conceptualizing them as transportable and socially relationally constructed. Although perspectives that emphasize physical presence and contact with such geographically bound definitions of place and land are most commonly recognized in both mainstream (72) and Indigenous literature (73), this current study adds an additional dimensionality that is relevant to increasing diverse, intersectional, and transnational (e.g., urban to rural or national to international) Indigenous populations around the world. Thus, these findings further articulate and add to the extant Indigenous literature to envision the broad possibilities of relational place-making as interventive spaces, much like the Native Hubs of Danza described in this article.

This study also warrants further investigation into understanding what other Native Hubs look like for populations like the UDMC across the world. Such research can inform policy initiatives that recognize the unique and crucial role that culture and place play in addressing Indigenous health disparities and inequities. With ever-increasing urbanization and migration of all peoples across the globe—Indigenous included—it is essential that policymakers recognize the importance of making funding streams available that accommodate the considerable financial resources and time needed to support the development of cultural and place/settings-based, community-based interventions as Native Hubs.

### Future Directions

This study illuminates pathways for culturally grounded intervention research development with the UDMC and similar populations. Its findings point to the potential of culturally grounded practices as proactive, primary prevention interventions to address risk for adverse health outcomes before they take root, thereby mitigating the health and fiscal consequences involved in rendering secondary- and tertiary-focused prevention interventions for those already exposed to risk or those who have become ill (74). Taking strengths-based, primary prevention approaches aligns with Indigenous communities' efforts to reduce stigmatizing narratives believed to be primarily reinforced by much of the extant research on risk factors within Native communities. Such culturally grounded interventions counter pathologizing narratives through promoting decolonizing narratives that draw on traditional health knowledge and practices. For participants in the UDMC, these go beyond the physical health benefits of participating in cultural dance to include other benefits that “can build connectedness, model positive social norms, personal wellness, and contribute to other positive outcomes” [(75) p. 2, (76, 77)] and have potential to serve as multilevel, community-level interventions that can positively impact overall community health and well-being in a sustainable way (78). Given the results from this study, in particular, publications highlighting the theoretical and methodological innovations and the substantive findings surrounding Danza as a Native Hub will help build the body of literature in this area for which there has previously been little empirical work.

The findings of this study translate into important future directions for developing prevention research in several ways. First, they shed light on the potential for theoretical development in recognizing Danza as a place to other, similar populations' unique cultural practices as potential, relationally constructed Native Hubs. Such theoretical development could provide a unique and innovative example of how culturally protective activities can be conceptualized as Native Hubs within a relational geography lens that builds on Indigenous principles of relationality and connectedness. Second, applying this study's qualitative, narrative methods approach to other UDMC samples would evaluate the transferability of the DNOH model as a general health model (see Figure 1) to other similar urban, transnational and diasporic Indigenous communities to determine its applicability. Furthermore, taking a qualitative and quantitative mixed-methods approach to conduct a longitudinal analysis of the impact of UDMC participation on changes in health attitudes and behaviors would additionally yield more robust data from which to assess its effectiveness among the sample populations. Third, these findings can be used to develop culturally appropriate measures to assess multilevel risk more accurately and protective factors appropriate for this vulnerable population (78). As tools for collecting data from larger samples, such measures could foreground the production of more generalizable findings that can be used to advocate for more funding toward culturally grounded interventions that serve urban, transnational, and diasporic Indigenous communities like the UDMC.

Second, the body of research on epigenetics is growing, particularly surrounding the transmission of intergenerational trauma (79). More work in this area with Indigenous populations could fill a critical gap in health research. Epigenetic and other scientists have also been calling for an increased investigation of the intergenerational transmission of resilience (79). However, such work should be approached with caution, given both the harmful, exploitative history of genetic (e.g., eugenics) and other health research in Indigenous and other marginalized communities (80) and the potential for policymakers to shift “the responsibility away from societal factors,” with awareness “that epigenetics could be used for racist agendas that work against Indigenous health and well-being” (81).

Third, there is also a clear need for greater integration and collaboration among more traditional, cultural practices. Such practices could include the use of *curanderos/as* (82) and/or other Indigenous healers. Furthermore, these practices can be integrated into and/or extend beyond the four walls of Western medical care facilities to therapeutic places and settings that are culturally relevant to marginalized communities, particularly for Indigenous and other communities whose identities and well-being are often intertwined with land and other than human relatives [e.g., plants, animals; (83)]. Practitioners, researchers, and policymakers should advocate for such collaborations and partnerships to build a more holistic, effective, and sustainable approach to care that aligns with the United Nations Permanent Forum on Indigenous Issues recommendations for governmental support of Indigenous health and resource centers (84). This study provides initial evidence that could support pathways toward additional qualitative, mixed-methods, and quantitative research that illuminates the impacts of place/settings-based, culturally grounded evidence on the prevention of health disparities and promotion of health and well-being.

### Limitations

This study has three major limitations. First, Mexican American Indians (MAI) is a new U.S. Census subcategory representing a diverse, complex, and intersectional population for which there is limited empirical data (12). Thus, we review health literature among AIAN and Latinx as proxy demographic groups in which MAIs may have previously been categorized before the U.S. Census Bureau developed the MAI subcategory. These bodies of literature comprise great diversity within and across groups. They offer essential available empirical insight at this time as to the status of health among UDMC as a population with potentially similar colonial-based historical and contemporary determinants of health. Furthermore, the extant epidemiological literature reveals considerable similarities in AOD and HIV risk across these groups, highlighting the need for empirical investigation of health risks and protective factors among those who may identify as MAIs.

Second, the transferability of the findings of this study is limited due to the small sample size. However, we aimed to obtain rich, in-depth, qualitative, narrative data that reveals essential, culturally relevant, and meaningful health implications for UDMC. Furthermore, this sample of UDMC comprises participants whose origins come from four specific Indigenous Peoples originating from northern, central, and southern states of Mexico, with a diversity of ages, educational backgrounds, languages spoken, and genders. Although the sample is small, it is diverse in several arenas, strengthening the potential for transferability of findings to other groups of UDMC or similar communities. Furthermore, methodological integrity—a conceptual framework of criteria for trustworthiness in qualitative research—was used to evaluate whether the researcher achieved *fidelity to the subject matter* and *utility in achieving goals* (85, 86). This aligns with culturally appropriate standards of rigor that reinforce Indigenous principles of relational accountability between the researcher and the community who provide their stories as data, reflected in theoretical, methodological, and analytical choices in response to the research question and concerning the participant community.

Finally, although the needs assessment and this specific study analyzed participant narratives associated with AOD and HIV risk and several other areas of health experiences were discussed (e.g., mental health, spiritual health, and nutrition), authors did not evaluate for health outcomes related to these conditions so we cannot provide empirical evidence of the effectiveness of Danza as a prevention or intervention strategy. Rather, the data spoke to participant experiences and perceptions of AOD, HIV and other health risks, which is a first step in identifying promising prevention or intervention approaches. More research is needed to evaluate and measure the impact of Danza Mexica on these health outcomes.

## Conclusion

This study illuminates how members of the UDMC are creating culturally grounded pathways for health and prevention for current and future generations through engaging in Danza Mexica as a Native Hub. Through innovative use of narrative methods to develop theory, shape methodological approaches, and synthesize stories as data, this study provides a unique opportunity to bear witness to the courage and strength of the UDMC to remember their ancestral pasts, shape their present, and envision their futures. The UDMC's narratives exemplify resilience despite historical and contemporary traumas, adapting to change in order to survive, and maintaining a continuous cultural thread to their past through current ceremonial practices. Indeed, their contemporary narratives *are* an expression of their emerging roots. For the UDMC, the practice of Danza Mexica creates community—a deep ancestral connection lived with ancestors (as both human and homelands) at the moment. Place becomes transportable and expands time and generations. The UDMC, like many complex, intersectional, marginalized communities, are actively defining their healing pathways within their Native Hubs, building on an ancient narrative that speaks to a challenging yet rich past and a rapidly evolving future. Their existence brings space, place, and time together in a way that ensures the health and well-being of future generations.

## Data Availability Statement

The raw de-identified data supporting the conclusions of this article will be made available by the authors, without undue reservation.

## Ethics Statement

The studies involving human participants were reviewed and approved by University of Washington and University of Denver Internal Review Boards. The patients/participants provided their written informed consent to participate in this study.

## Author Contributions

This paper originates from AF's doctoral dissertation. AF developed the research question, conducted the analysis, and wrote the manuscript. RB is the principal investigator of the parent study, and served as a committee member for AF's dissertation. RB provided guidance, feedback, and edits to AF throughout the design, analysis, and writing of the article. All authors contributed to the article and approved the submitted version.

## Funding

Research reported in this publication was supported by the National Institute of Mental Health of the National Institutes of Health under Award Number R25MH084565, which funded the parent study. The National Institute of General Medical Sciences of the National Institutes of Health under Award Number S06GM127164 provided partial support for the AF's dissertation work.

## Author Disclaimer

The content is solely the responsibility of the authors and does not necessarily represent the official views of the National Institutes of Health or the Substance Abuse and Mental Health Services Administration.

## Conflict of Interest

The authors declare that the research was conducted in the absence of any commercial or financial relationships that could be construed as a potential conflict of interest.

## Publisher's Note

All claims expressed in this article are solely those of the authors and do not necessarily represent those of their affiliated organizations, or those of the publisher, the editors and the reviewers. Any product that may be evaluated in this article, or claim that may be made by its manufacturer, is not guaranteed or endorsed by the publisher.
